# Predictive value of C-reactive protein in patients treated with sunitinib for metastatic clear cell renal cell carcinoma

**DOI:** 10.1186/s12894-017-0267-6

**Published:** 2017-08-31

**Authors:** Martin Pilskog, Christian Beisland, Lars A. Akslen, Leif Bostad, Åse Haug, Daniel Heinrich, Karin M. Hjelle, Oddbjørn Straume

**Affiliations:** 1 Centre for Cancer Biomarkers CCBIO, University of Bergen, Bergen, Norway; 2 Department of Oncology, Haukeland University Hospital, 5021 Bergen, Norway; 3 Department of Urology, Haukeland University Hospital, 5021 Bergen, Norway; 4 Department of Pathology, Haukeland University Hospital, 5021 Bergen, Norway; 5 Department of Clinical Medicine, University of Bergen, Bergen, Norway; 6 Department of Oncology, Akershus University Hospital, 1478 Lørenskog, Norway

## Abstract

**Background:**

Sunitinib has become mainstay first line treatment for patients with metastatic renal clear cell carcinoma (mRCC). Still, useful predictive markers of response are lacking and urgently needed for clinical decision making.

**Methods:**

In the present study we investigated the predictive value of standard serum markers as well as clinical markers, including C-reactive protein (CRP), Neutrophil to Lymphocyte ratio (NLR) and early hypertension (eHTN) in an unselected prospective patient population treated with sunitinib for mRCC. Forty-six patients were enrolled in a prospective single-arm study of predictive markers for sunitinib response. Response rates according to RECIST 1.1 were used as primary end-point. Secondary objectives were to evaluate prognostic value of the candidate markers with regard to progression free survival (PFS) and overall survival (OS). In addition, toxicity rates and quality of life was recorded.

**Results:**

Median PFS and OS was 9.1 months and 15.0 months, respectively. Of 38 patients evaluable for response, 1 patient had complete response (CR), 7 had partial response (PR), 18 had stable disease (SD) and 12 had progressive disease (PD). Normal CRP at baseline was significantly associated with objective response (CR + PR) (*p* = 0.01). Normal CRP was also significantly associated with improved PFS and OS (Log rank, *p* = 0.05 and <0.01, respectively). Early hypertension, NLR and IMDC risk score were not significantly associated with response rates or survival.

**Conclusion:**

Baseline CRP was a significant predictive factor of sunitinib response *and* a prognostic factor of survival. Baseline CRP might be a useful biomarker in the treatment planning of mRCC. Due to the relatively small sample size, our results need to be confirmed in larger studies.

**Electronic supplementary material:**

The online version of this article (10.1186/s12894-017-0267-6) contains supplementary material, which is available to authorized users.

## Background

The frequent inactivation of the Von Hippel Lindau (*VHL*) gene in clear cell renal cell carcinoma, leading to increased levels of hypoxia inducible factor 1 (HIF1) and vascular endothelial growth factor (VEGF), provides the rationale for treatment with antiangiogenic receptor tyrosine kinase (rTKI) inhibitors. Since the reporting of the first positive clinical trial [1], showing an overall survival benefit from a rTKI, sunitinib has become mainstay first line treatment for patients with metastatic renal cell carcinoma (mRCC). Although objective response rates are reported for around 50% of the patients, development of resistance to the treatment is a major problem [2]. Clearly, a subset of patients does not benefit from treatment with sunitinib, and side effects are frequent. Interestingly, hypertension is a common side effect of angiogenesis inhibitors and has been associated with improved treatment response [3]. In the research community, considerable effort has been made to identify and validate predictive biomarkers of response to sunitinib treatment, but so far, no biomarkers have been established as useful in clinical decision making and treatment planning.

There is increasing evidence to support an important role of systemic inflammation in development and progression of RCC [4], as recently substantiated by positive results from a clinical trial with the PD-1 inhibitor nivolumab in mRCC [5]. VEGF does not only stimulate tumor associated angiogenesis, but also plays an important role in the local immune response in wounds (physiologic) and tumors (pathologic) by inducing accumulation of immature dendritic cells, myeloid-derived suppressor cells, regulatory T cells, as well as by inhibiting the migration of T lymphocytes to the tumor [6]. Thus, it is relevant to also explore biomarkers primarily associated with inflammatory responses in the search for predictive markers for response to anti-VEGF therapy.

C-reactive protein (CRP) is an established biomarker for systemic inflammation, available in most clinical datasets, and provides prognostic information in several cancers including RCC [7]. Another biomarker of inflammation, neutrophil-to-lymphocyte ratio (NLR), adds prognostic information in RCC, and was recently suggested as a predictive marker of response to sunitinib in mRCC [8]. In the present trial we investigated the predictive value of serum markers, including CRP and NLR, in an unselected prospective patient population treated with sunitinib for mRCC. In addition, we report on toxicity and health related quality of life (HRQoL) data.

## Methods

### Patients and treatment

Between October 2007 and October 2014, a regional cohort of 77 patients with mRCC was screened for inclusion in this prospective study at Haukeland University Hospital, Bergen, Norway. Forty-six patients were enrolled after signing the informed consent sheet (CONSORT Flow Diagram, Additional file 1: Fig. S1). Inclusion criteria included: previously untreated metastatic or non-resectable clear cell RCC, WHO performance state 0–2, no known brain metastases, evaluable tumor lesions according to RECIST (version 1.1) and no significant comorbidity or laboratory abnormalities. See Additional file 2: Table S1 for all inclusion criteria. Sunitinib was administered 50 mg/day on schedule 4 weeks on/ two weeks off. Patients continued on treatment until disease progression, significant toxicity or consent withdrawal. Data was collected from the hospital records and included demographics, treatment modifications, adverse events, radiologic response data and survival. Data cut-off date was July 31 2015.

The main objective of this study was to identify and evaluate the predictive value of candidate markers readily available in a standard clinical setting, in mRCC patients treated with sunitinib. Candidate markers included early hypertension (eHTN), IMDC risk groups, baseline neutrophil to lymphocyte ratio (NLR), baseline CRP and baseline EORTC QoL symptom scale. Response rates according to RECIST 1.1 were used as primary endpoint. Secondary objectives were to evaluate prognostic value of the candidate markers with regard to progression free survival (PFS) and overall survival (OS). In addition, toxicity rates and HRQoL was recorded.

### Assessment of response, adverse events and quality of life

The primary endpoint was objective response (OR) defined as complete response (CR) or partial response (PR) according to RECIST v.1.1 as well as clinical benefit (CB) defined as CR + PR and including stable disease (SD) for more than 6 months. Disease stabilization is considered beneficial to patients experiencing progression at the time of inclusion and CB is frequently included as an additional statistical endpoint in trials investigating antiangiogenic drugs in which therapeutic activity and clinical benefit are present, even in the absence of radiological tumor shrinkage [9]. Importantly, all patients were in clinical and/or radiological progression at the time of inclusion. OR and CB were calculated on the basis of investigator assessment. Response evaluation by CT-scan or MRI was performed every 12 weeks. Patients with clinically evident disease progression or death due to mRCC before first radiological progression were recorded as progressive disease (PD). Best overall response (BOR), recorded as change in size of target lesion, was not available in these patients. PFS was defined as the time from treatment initiation until disease progression according to RECIST v.1.1. OS was defined as the time from enrollment until death of any cause.

Standard blood samples, including CRP and neutrophil/lymphocyte counts, were taken at treatment initiation and every 6 weeks during treatment. Adverse events were graded according to the National Cancer Institute Common Terminology Criteria for Adverse events, version 3.0 (CTCAE v.3.0), and were recorded at each 6-week cycle. Early hypertension (eHTN) was particularly evaluated for its potential role as a predictive marker for treatment response. We recorded eHTN in two different ways. First, we defined eHTN as either maximum post-baseline systolic blood pressure (SBP) ≥140 mmHg or maximum post-baseline diastolic blood pressure (DBP) ≥90 mmHg recorded at week 6 and week 12 [3]. Second, we recorded eHTN as HTN ≥ grade 1 defined by CTCAE v.3.0 at week 6 and week 12. All other adverse events were recorded every six weeks throughout the entire treatment period. Patients that stopped treatment due to toxicity before 1st tumor response evaluation at week 12 were not included in the analyses of response rates or PFS, but were included in the analyses of OS.

HRQoL was assessed by a validated Norwegian version of the questionnaire of the European Organization for Research and Treatment of Cancer (EORTC QLQ-C30 v.3.0) at baseline and every 12 weeks during treatment. The QLQ-C30 contains a global health/QoL scale, five functional scales (physical, role, cognitive, emotional, and social), three symptom scales (fatigue, pain, and nausea/vomiting) and six single items (dyspnoea, insomnia, anorexia, constipation, diarrhoea, and financial difficulties). The answers are given according to a 4-point Likert format, with the exception of questions about general health and quality of life, which are given according to a 7-point Likert format. Scores was calculated as described in the EORTC QLQ-C30 Scoring manual (3rd edition) [10]. The C 30 functional scales and the global scale were transformed so that 100% indicates best function and 0% least function of the individual QoL index, whereas the C30 symptom scales were transformed so that 0% indicates the least and 100% the most symptoms. We compared the upper quartile with the lower 3 quartiles for the symptom sum score and the lower quartile with the upper 3 quartiles for the functional sum score and global health/QoL score.

### Statistical analyses

The Mann-Whitney U test was used to compare the distribution of continuous variables between two groups such as responders and non-responders. Comparisons between categorical variables were performed by using the Fisher’s exact test. Spearman’s rank correlation coefficient was used to test correlations between variables of interest. Logistic regression analysis was used to test the relative importance of predictive factors for sunitinib response. Cronbach’s α was used to estimate the reliability of the global health score and the functional/symptomatic scores made up of more than one question. Kaplan-Meier estimates were applied for time-to-event endpoints such as PFS and OS, and log rank-test was applied for testing of differences between groups. Sample size calculations were based on a difference in response rate of 40% (i.e. 10% and 50%) between groups identified by the candidate markers. Thirty-eight patients were needed to achieve a power of 80% with an α-value of 0.05. All *p*-values are two-sided. Statistical investigation were done using IBM SPSS Statistics version 22.

## Results

### Patient population and treatment efficacy

The characteristics of the 46 patients enrolled in the study are presented in Table 1. In our cohort the median age was 63.1 (range 41.1–84.0). By July 31st, 2015 the median follow up time was 13.8 months (range 1.8–83.9). Twenty-six patients had prior removal of the primary tumor, twenty-four by radical and 2 by partial nephrectomy. Six patients had resection of bone metastasis, eight patients had resection of other metastasis, one patient had gamma knife radiosurgery of brain metastasis and two patients had radiation therapy against bone metastasis prior to sunitinib treatment. Median time on treatment was 5.7 months (range 0.5–63.0). Median time from first diagnosis of renal cell carcinoma to treatment was 3.2 months (range 0.3–124). Median time to treatment from diagnosis of metastasis was 1.4 months (range 0.3–66.5).

**Table 1:** Baseline Patients Characteristics

	Study cohort (*n* = 46)
Age, years	
Median	63.1
Range	41.1–84.0
Sex - No. (%)	
Male	29 (63.0)
Female	17 (37.0)
WHO performance status - No. (%)	
0	30 (65.2)
1	16 (34.8)
2	0 (0.0)
Site of metastases - No. (%)	
Brain	1 (2.2)
Lung	35 (76.1)
Pleura	3 (6.5)
Liver	4 (8.7)
Bone	16 (34.8)
Lymph nodes	28 (60.9)
Number of disease sites - No. (%)	
1	10 (21.7)
2	11 (23.9)
≥ 3	25 (54.3)
Hypertension before treatment - No. (%)	
Yes	24 (52.2)
No	22 (47.8)
IMDC risk score – No. (%)	
Good	7 (15.2)
Intermediate	16 (34.8)
Poor	21 (45.7)
*Missing*	*2 (4.3)*
Time from initial diagnosis - No. (%)	
≤ 12 months	33 (71.7)
> 12 months	13 (28.3)
Prior removal of primary tumor - No. (%)	
Radical nephrectomy	24 (52.2)
Partial nephrectomy	2 (4.3)
No	20 (43.5)

*Abbreviations*: *WHO* World Health Organisation, *IMDC* International Metastatic Renal Cell Carcinoma Database Consortium

By July 31 2015, median progression free survival (PFS) was 9.1 months (range 0.5–57.3) and median overall survival (OS) was 15.4 months (range 1.8–83.9). At data cut-off, 9 patients were still alive, and six patients were still on sunitinib treatment without signs of progression. Twenty-three patients started second line systemic treatment. We observed 1 complete response (CR), 7 partial responses (PR) and 18 patients had stable disease (SD) ≥ 6 months. Twelve patients showed progressive disease (PD), of which 10 were confirmed by radiology and 2 were confirmed by clinical progress before week 12. Eight patients stopped treatment before week 12 and were recorded as non-evaluable for response rates and PFS. Of these, six were due to toxicity without evidence of disease progression, one patient due to appendicitis and one protocol violation. Of interest, seven of these eight patients were females.

### Predictive value of pre-treatment clinical and biochemical markers and survival analyses

The correlations between clinical, as well as biochemical markers assessed ahead of treatment initiation and sunitinib response are given in Table 2. The association between clinical, as well as biochemical markers assessed ahead of treatment initiation, PSF and OS is given in Table 3.

**Table 2:** Univariate analyses of clinical and biochemical markers in relation to response to sunitinib

Variable	Best overall tumor response (RECIST ver. 1.1)					
	OR^1^ n(%)	SD^2^+PD^3^ n(%)	*p* value^4^	CB^5^ n(%)	PD^3^ n(%)	*p* value
Total	8(21)	30(79)		26(68)	12(32)	
Age			0.69			0.73
< 63.1	4(17)	19(83)		15(65)	8(35)	
≥ 63.1	4(27)	11(73)		11(73)	4(27)	
Sex			0.17			0.45
Female	4(40)	6(60)		8(80)	2(20)	
Male	4(14)	24(86)		18(64)	10(36)	
Number of disease sites			0.71			0.31
≤ 2	3(18)	14(82)		10(59)	7(41)	
> 2	5(24)	16(76)		16(76)	5(24)	
Prior nephrectomy			0.26			0.73
Yes	6(29)	15(71)		15(71)	6(29)	
No	2(12)	15(88)		11(65)	6(35)	
Pretreatment hypertension^6^			0.70			0.30
Yes	5(26)	14(74)		15(79)	4(21)	
No	3(16)	16(84)		11(58)	8(42)	
Treatment induced eHTN^7^ ≤ week 6			1.00			0.70
Yes	4(23)	13(77)		13(76)	4(24)	
No	4(27)	11(73)		10(67)	5(33)	
Treatment induced eHTN^8^ ≤ week 12			0.53			0.04
Yes	5(33)	10(67)		13(87)	2(13)	
No	0(0)	4(100)		1(25)	3(75)	
IMDC risk			0.77			0.46
Good	2(29)	5(71)		6(86)	1(14)	
Intermediate	3(25)	9(75)		9(75)	3(25)	
Poor	3(18)	14(82)		10(59)	7(41)	
NLR baseline ≤3			1.00			0.46
Yes	5(23)	17(77)		16(73)	6(27)	
No	3(25)	9(75)		7(58)	5(42)	
NLR week 6 ≤ 3			0.32			0.15
≤ 3	7(23)	23(77)		22(73)	8(27)	
> 3	0(0)	6(100)		2(33)	4(67)	
NLR shifted from >3 to ≤3 at week 6			1.00			0.06
Yes	2(25)	6(75)		6(75)	2(25)	
No	0(0)	3(100)		0(100)	3(100)	
NLR shifted from ≤3 to >3 at week 6			1.00			0.50
Yes	0(0)	2(100)		1(50)	1(50)	
No	5(26)	14(74)		14(74)	5(26)	
CRP ≤10 (mg/L)			0.01			0.09
Yes	7(41)	10(59)		14(82)	3(18)	
No	1(5)	19(95)		11(55)	9(45)	
EORTC QoL symptom scale at BL^9^			0.31			0.02
Upper quartile	0(0)	7(100)		2(29)	5(71)	
Lower 3 quartiles	8(27)	22(73)		24(80)	6(20)	

*Abbreviations*: *IMDC* International Metastatic Renal Cell Carcinoma Database Consortium, *LDH* Lactate dehydrogenase, *ULN* Upper limit of normal, *LLN* lower limit of normal, *NLR* neutrophil/lymphocyte ratio, *CRP* C-reactive protein, *BL* baseline

^1^Objective response (Complete + Partial response)

^2^Stable disease

^3^Progressive disease

^4^Fisher’s exact test

^5^Clinical benefit (OR + SD)

^6^Defined as on anti-hypertensive treatment before initiation of sunitinib

^7^Defined as systolic blood pressure(SBP) ≥140 mmHg or diastolic blood pressure (DBP) ≥90 mmHg ≤ week 6

^8^Defined as ≥140 mmHg or diastolic blood pressure (DBP) ≥90 mmHg ≤ week 12

^9^Quality of Life

**Table 3:** Survival analyses according to clinical and biochemical variables

Variable	PFS^1^			OS^2^		
	Median	95% CI^3^	*p*-value^4^	Median	95% CI	*p*-value
Age			0.29			0.47
< 63.1	8.7	6.2–11.2		17.5	5.5–29.4	
≥ 63.1	20.4	3.2–37.7		15.0	12.4–17.6	
Sex			0.03			0.87
Female	NR	-		12.7	7.3–18.2	
Male	8.7	6.6–10.7		18.0	10.5–25.4	
Number of disease sites			0.80			0.52
≤ 2	12.9	2.1–23.7		17.5	11.3–23.7	
> 2	9.1	8.3–9.8		13.9	10.2–17.6	
Prior nephrectomy			0.07			<0.01
Yes	14.7	5.9–23.5		26.0	20.1–31.8	
No	8.7	3.1–14.3		10.8	4.5–17.0	
Pretreatment hypertension			0.42			0.79
Yes	17.0	6.2–27.7		18.0	9.4–26.5	
No	8.4	3.7–13.1		11.6	5.3–17.9	
Treatment induced early hypertension^5^ at week 6			0.68			0.85
Yes	14.7	9.6–19.8		18.0	3.5–32.5	
No	8.7	3.9–13.5		12.1	3.8–20.4	
Treatment induced early hypertension^5^ at week 12			<0.01			<0.01
Yes	14.7	10.1–19.3		26.0	24.1–27.9	
No	2.6	1.9–3.3		7.7	4.4–11.0	
IMDC risk score			0.10			<0.01
Good	20.4	13.1–27.7		67.9	38.4–97.5	
Intermediate	9.1	6.0–12.2		12.7	10.6–14.9	
Poor	8.4	0–17.7		13.7	5.4–22.1	
NLR baseline ≤3			0.05			0.06
Yes	14.7	8.8–20.6		25.2	10.6–39.8	
No	6.7	2.0–11.4		13.2	10.3–16.1	
NLR week 6 ≤ 3			0.09			<0.01
Yes	10.8	6.0–15.7		25.2	13.7–36.7	
No	1.8	0.2–3.5		3.8	3.3–4.3	
NLR shifted from >3 to ≤3 week 6			<0.01			<0.01
Yes	8.4	6.0–10.7		13.2	7.0–19.4	
No	1.3	0.9–1.7		3.6	0.8–6.4	
NLR shifted from ≤3 to >3 at week 6			0.75			0.16
Yes	NR	-		4.0	2.3–5.7	
No	14.7	9.2–20.3		26.0	24.2–27.7	
CRP			0.05			<0.01
≤ 10 (mg/L)	14.7	2.5–26.9		26.0	0.6–51.4	
> 10 (mg/L)	5.3	0.9–9.8		12.1	8.8–15.5	
EORTC QoL symptom scale at BL			<0.01			0.01
Upper quartile	2.8	2.2–3.5		12.7	4.8–20.7	
Lower 3 quartiles	14.7	4.6–24.8		25.2	12.7–37.7	

*Abbreviations*: *IMDC* International Metastatic Renal Cell Carcinoma Database Consortium, *LDH* Lactate dehydrogenase, *ULN* Upper limit of normal, *LLN* lower limit of normal, *NLR* neutrophil/lymphocyte ratio, *CRP* C-reactive protein, *NR* Not reached, *BL* Baseline

^1^Progression free survival

^2^Overall survival

^3^Confidence interval

^4^Log rank test

^5^Defined as systolic blood pressure(SBP) ≥140 mmHg or diastolic blood pressure (DBP) ≥90 mmHg

### C-reactive protein (CRP)

**Fig. 1:**
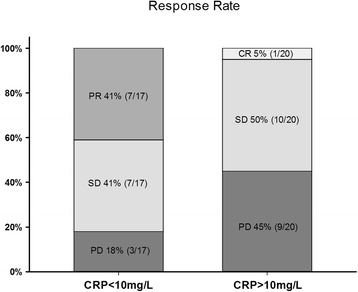
Response rates as a function of baseline CRP levels. Patients with a baseline CRP ≤ 10 mg/L (normal) showed an objective response rate (CR + PR) of 41% whereas patients with elevated CRP showed an objective response rate of 5%. CR, complete response; PR, partial response; SD, stable disease; PD, progressive disease

**Fig. 2:**
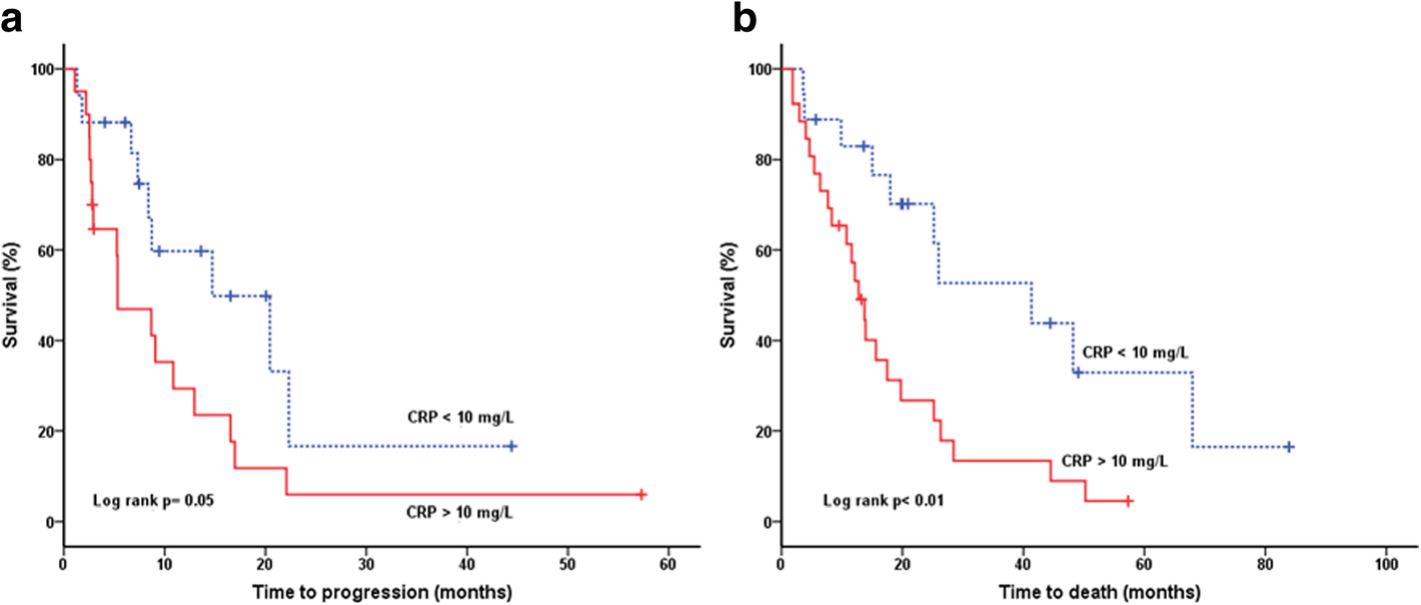
Kaplan-meier estimates of **a**) progression free survival (PFS) and **b**) overall survival (OS) grouped by CRP level. The normal CRP (≤ 10 mg/L) cohort showed significantly improved PFS and OS compared with patients with elevated CRP

Median CRP at baseline was 17.0 mg/L, range 0–235 mg/L. Seventeen of 37 patients evaluated for overall response had normal CRP (≤10 mg/L). Normal CRP at baseline was significantly associated with OR (CR + PR) (Fisher’s exact test, *p* = 0.01) (Fig. 1). Seven/17 (41%) of patients with normal CRP had an objective response to sunitinib, compared with 1/20 (5%) patients with elevated CRP had an objective response. Logistic regression analysis was used to test the relative importance of the candidate predictive factors for sunitinib response(eHTN, IMDC risk groups, baseline NLR, baseline CRP, baseline EORTC QoL symptom scale). Only CRP level at baseline was an independent predictive variable of response, with an odds ratio of 14.3 (*p* = 0.02) of not having an objective response if CRP was above normal (10 mg/L). CRP at baseline was significantly correlated with several other variables including age, function sum score, symptom sum score, performance status and tumor load (Additional file 3: Table S2). Median PFS was significantly longer among patients with normal CRP at baseline (median 14.7 vs 5.3 months, log rank *p* = 0.05). Similarly, an improved OS was found in patients with normal CRP at baseline (median 26.0 vs 12.1 months, log-rank *p* < 0.01) (Fig. 2 a, b).

### Neutrophil to lymphocyte ratio (NLR)

Twenty-two of 34 (64%) patients evaluated for response and available NLR, had NLR ≤3 at baseline (median = 2.7, range 1.0–7.9) and NLR at baseline was not significantly correlated with OR or CB. Eighty-three % of patients evaluated for response had NLR ≤3 at week 6 (median = 1.6, range 0.4–5.9) and this was not significantly correlated to OR or CB. A shift from NLR >3 at baseline to ≤3 at week 6 (*n* = 10) was not significantly associated with OR or CB. Median PFS among patients with baseline NLR ≤3 was significantly better than patients with baseline NLR >3 (median 14.7 vs 6.7 months, log rank *p* = 0.05). A borderline association was present between baseline NLR ≤3 and OS (median 25.2 vs 13.2 months, log-rank *p* = 0.06). High NLR at baseline was significantly correlated with increased tumor load (*r* = 0.43, *p* = 0.005, Spearman).

### Treatment induced early hypertension (eHTN)

Applying the first definition of eHTN (SBP ≥140 or DBP ≥90 mmHg at week 6 and week 12) seventeen of 32 patients (53%) evaluated for response had eHTN after week 6. Median SBP over DBP was 145 (range: 120–170) mmHg over 89 (range: 60–170) mmHg, and was not significantly associated to OR or CB. Using the same definition at week 12, fifteen of 19 patients (79%) had eHTN. Median SBP over DBP was 142 (range: 120–170) mmHg over 88 (range: 65–107) mmHg, and was significantly associated with improved CB, but not OR (Fischer’s exact test *p* = 0.04 and *p* = 0.53, respectively)(Table 2). The second definition (based on CTCAE v 3.0) of eHTN was not significantly associated OR or CB (data not presented). eHTN at week 12 was associated with improved PFS and OS (Table 3). All seven patients with increased blood pressure during the two first cycles used anti-hypertensive drug(s) at baseline.

### Risk scores

The distribution of IMDC risk score [11] is given in Table 1. IMDC risk score was not significantly associated with OR or CB. Good IMDC risk score versus intermediate and poor was not significantly correlated to PFS (median 20.4 vs 9.1 vs 8.4 months, log-rank *p* = 0.10), but was significantly associated with OS (median 67.9 vs 12.7 vs 13.7 months, log-rank *p* = <0.01). We found similar results for MSKCC risk score and WHO performance status (PS) (data not presented).

### Metastatic sites

The distribution of metastatic sites is given in Table 1. There was no significant association between metastatic site and response rates. Although present in only 3 patients, pleura metastasis was significantly associated with reduced PFS (median 2.6 vs 9.1 months, log rank *p* = 0.05) and OS (median 10.8 vs 17.5 months, log-rank *p* = 0.04). Presence of lung metastasis was significantly associated with reduced OS (median 13.2 vs 48.2 months, log-rank *p* = 0.04). Other metastatic sites (brain, bone, liver or lymph nodes) were not significantly associated with PFS or OS.

### Toxicity

Adverse events occurring during treatment with sunitinib according to CTCAE v.3.0 are summarized in Additional file 4: Table S3. The most common adverse effects of lower grade (1 + 2) were nausea (52.2%), anemia (47.8%), fatigue (45.7%) and diarrhea (39.1%). The most common severe adverse effects (grade 3 + 4) were hypertension (19.6%), fatigue (15.2%), low serum platelets (15.2%), hand-foot skin reaction (10.9%) and diarrhea (10.9%). We observed one grade 5 adverse effect (death due to appendicitis) probably not related to sunitinib treatment.

### Health related quality of life

The results of the HRQoL questionnaires at baseline (*n* = 45) and at the first treatment evaluation (*n* = 28, after 12 weeks) are presented in Additional file 5: Table S4. Only for “Fatigue”, there was a statistically significant increase in the score during treatment compared with the baseline value (*p*=0.041, Wilcoxon ranked signed test). The Cronbach- α of the indices derived by more than one question showed acceptable/good values (0.74-0.89), except for “cognitive function” (0.30) and “social functioning” (0.56). The Cronbach- α of the sum scores of functional indexes (0.80) and symptom indexes (0.79) were acceptable/good. In contrast to Global health status/QoL and Functional scale, a Symptom sum score below median was significantly associated to improved CB (Fisher’s exact test, *p* = 0.02, Table 2). Investigating symptom sum scores indicated that the upper quartile had significantly worse OS (median 12.7 vs 25.2 months, log-rank *p* = 0.01) and PFS (median 2.9 vs 14.7 months, log-rank *p* = <0.01). No such difference could be demonstrated for global health/QoL status or functional sum score (data not shown).

## Discussion

Until recently, palliative surgery, radiation therapy and chemotherapy were the only treatment options for metastatic RCC (mRCC), and primary therapy resistance, reduced quality of life and short survival were major challenges in this patient group. Currently, three major categories of systemic treatment exist for the largest subgroup of mRCC, the clear cell carcinomas: cytokines and immune checkpoint inhibitors, anti VEGF targeted drugs and mTOR inhibitors [12]. The two latter of these new treatment options have emerged based on recent knowledge of the pathogenesis of clear cell renal cancer. The von Hippel-Lindau (VHL) tumor suppressor gene is lost or mutated in 60–90% in sporadic cases [13] and is a major contributor to development of this cancer. Loss of VHL leads to a chronic stress response state in the cells trough high levels of HIF1-α, a transcription factor for a number of stress response proteins, including vascular endothelial growth factor (VEGF). In addition to being a potent angiogenic growth factor, VEGF plays a role in the local immune response in wounds and tumors by inducing accumulation of immature dendritic cells, myeloid-derived suppressor cells, regulatory T cells, as well as by inhibiting the migration of T lymphocytes to the tumor [6]. Renal cell cancer is regarded as highly immunogenic and angiogenic tumors, supporting VEGF as a promising target for treatment. The VEGF receptor inhibitor Sunitinib is currently first line treatment for mRCC [12], but a significant portion of the patients do not respond, and the search for good predictive markers of response has been disappointing so far. Whereas most focus in the search for predictive markers has been on angiogenesis, less focus has been on markers of immune responses. In the current study, we evaluated readily available clinical and biochemical markers, associated with systemic inflammation, for their association with response to sunitinib.

C-reactive protein (CRP) is an acute-phase protein that increases rapidly following interleukin-6 secretion by macrophages and T cells following infection, inflammation and cancer [14]. CRP is a negative prognostic marker in most cancers. In the present study we found normal CRP to be a possible predictive factor of response. Whereas 41% of the patients with normal CRP at baseline experienced an objective response, this was the case for only 5% of patients with CRP levels at baseline above normal. CRP was also associated with PFS and OS supporting its role as a prognostic marker as well, and this is in line with previous reports [15–17]. Our finding supports the results of a recent study by Fujita et al. where normal level of CRP at baseline was an independent predictive marker of response by multivariate analysis [15]. In a retrospective study of 200 patients treated with sunitinib 61% of patients with normal CRP responded vs 32% of patients with elevated CRP [18]. In our trial, CRP was correlated to several factors including other markers of systemic inflammation such as high platelet counts, anemia as well as tumor load and performance status. Thus, CRP might represent a marker of disease burden identifying a patient subpopulation with poor prognosis, less likely to respond. Nevertheless, the significant association with response rates suggests that CRP might be a useful marker, in addition to other clinical and biochemical features to consider prior to initiation of systemic treatment. IL-6 is an important tumor-promoting protein associated with stress responses, inflammation and angiogenesis [19]. Through its major downstream target STAT3 several tumor promoting pathways are activated, including HIF1-α and VEGF [19]. Moreover, IL6 have direct stimulating effect on endothelial cells, and has been implicated in resistance to anti-VEGF therapy [20]. Being closely correlated to IL6 expression, increased CRP levels might therefore be a surrogate marker of IL6 driven disease, again being associated with expression of multiple angiogenic factors [21], thus less responsive to specific anti-VEGF treatment like sunitinib. Our results indicate that an inflammatory response, defined by high CRP is associated with poor response to sunitinib and poor prognosis in these patients. The effect of sunitinib on inhibiting the angiogenesis supporting and immunosuppressive effect of VEGF, thus seem to be more pronounced in patients with a non-inflammatory state defined by normal CRP.

The neutrophil-to-lymphocyte ratio (NLR) is also a marker of systemic inflammation in cancer patients and was found to add prognostic [22] and predictive [8] information in RCC in retrospective studies. Like CRP, NLR is readily available in standard blood samples in a regular clinical setting. Our NLR counts were comparable to what has been reported in other clinical datasets. Although significantly associated with CRP, we did not find a statistically significant association with sunitinib response or survival. The significant correlation with performance status and tumor load suggests that NLR is a nonspecific marker of disease burden. Still, due to relatively small sample size and low statistical power, our data must be interpreted carefully.

Treatment induced early hypertension (eHTN) was not significantly associated with treatment response in our dataset. In our patient population, the baseline blood pressure was slightly higher when compared with the clinical trial population studied by Rini et al. [3], and 52% of our patients were hypertensive at baseline. Still, the number of patients recorded as having sunitinib induced eHTN after cycle 1 and 2, using the same criteria, was nearly the same (~80%). eHTN at week 12 was associated with improved survival, but this is most likely due to the fact that the responders in the study stayed on treatment long enough to develop hypertension. Even if pharmacodynamically interesting, as eHTN occurs *after* sunitinib initiation it is not going to be an applicable predictive marker in the clinic.

Our finding that higher baseline HRQoL symptoms score is prognostic for PFS and OS in treatment with Sunitinib is in line with the earlier report by Cella et al. [23]. In that report, however, a different QoL tool was used. Herrmann et al. demonstrated by using EORTC QLQ-C30, that “global QoL” was prognostic for PFS [24]. Our study did not confirm this finding. In general, there were only small changes in HRQoL scores form baseline to 12 weeks. Herrmann et al. also showed a relatively small change in the different HRQoL scales after 12 weeks [24]. This could be due to the administration of Sunitinib (4 weeks on/ 2 weeks off), with subsequent remission of eventual treatment induced symptoms. There are indications that long-term survivors might retain a good HRQoL over years, as described by Carmichael et al. [25].

A major challenge in studies exploring predictive markers of treatment response in clinical data-sets is the fact that most of the candidate predictive markers are prognostic as well, thus significantly correlated with PFS and OS independent of the treatment given. Combined predictive and prognostic markers are best evaluated in two-arm trials. In single arm trials such as ours, response rates according to RECIST are superior to PFS and OS as primary end-point when assessing predictive markers of treatment response. Many biomarker studies in mRCC have been performed retrospectively in data-sets from large clinical trials, and these patients are frequently positively selected and do not optimally reflect the normal patient population. The strength of our study is the prospective design and the “Real-World” patient population enrolled, reflecting a normal clinical setting. When compared with large retrospective multicenter studies as well as smaller single-center studies, the majority of our patients were in the poor risk group according to IMDC criteria. Whereas the portion of poor-risk patients varies between 18 and 33% in comparable studies [8, 11, 15], 46% of our patients belonged to this group. In addition, all patients were in confirmed clinical and/or radiological progression at the time of inclusion. Accordingly, patients with very slow progression or stable metastases were observed without systemic treatment and screened for inclusion in the study only after confirmed disease progression. In comparison with clinical phase III trials, PFS and OS were lower in our patients. Compared with the adverse events reported in clinical trials [26], the frequency of toxicity from sunitinib in metastatic renal cell cancer recorded in our study was similar, or somewhat less frequent. Especially, the hematological toxicity including anemia, neutropenia, thrombocytopenia and lymphopenia was less frequent in our trial, although using the same criteria (CTCAE v. 3.0). The most likely explanation for this discrepancy is that we assessed adverse events, including laboratory, every 6 weeks, where most of the patients were off the drug in the 4 + 2 weeks cycle.

In addition to the lack of a control group, our study has some weaknesses. First, the number of patients included is low and thereby the study lacks the significant power to detect minor differences in response rates between groups based on the biomarkers under investigation. Thus, our finding should be validated in an independent and larger cohort of patients. Second, CRP and NLR are non-specific markers of inflammation and angiogenesis, and further studies are required to identify the key regulators controlling the systemic responses to metastatic disease. In this report, we focused on biomarkers available in standard clinical blood samples routinely used in the clinic. Further studies of candidate biomarkers in serum and plasma, such as IL6 and IL8 are ongoing.

## Conclusion

In conclusion, in this prospective study of sunitinib in patients with mRCC we found that normal level of s-CRP at baseline is significantly associated with improved response rates and might serve as guidance in the selection of optimal treatment. Still, due to the relatively small sample size and low statistical power, our results need to be confirmed in larger studies.

## Additional files

Additional file 1: Fig. S1. CONSORT 2010 Flow Diagram. (DOCX 36 kb)

Additional file 2: Table S1. Inclusion criteria. (DOCX 17 kb)

Additional file 3: Table S2. Correlations between CRP and other variables. (DOCX 15 kb)

Additional file 4: Table S3. Adverse effects. (DOCX 20 kb)

Additional file 5: Table S4. Summary of quality of life (QoL) scores. (DOCX 14 kb)

## Abbreviations

IMDC: International Metastatic Renal Cell Carcinoma Database Consortium
MSKCC: Memorial Sloan Kettering Cancer Center
