# Supplementary material for: Predictive value of C-reactive protein in patients treated with sunitinib for metastatic clear cell renal cell carcinoma
Source: BMC Urol. 2017 Aug 31;17:74. doi: 10.1186/s12894-017-0267-6 (PMC5580299; doi:10.1186/s12894-017-0267-6)
Supplement: Supplementary file 1 — CONSORT 2010 Flow Diagram. (DOCX 36 kb) [file 12894_2017_267_MOESM1_ESM.docx]

**CONSORT 2010 Flow Diagram**

**Figure S1.** 77 patients with metastatic renal cell carcinoma were screened for inclusion in the prospective clinical study. 46 patients were enrolled after signing the informed consent sheet. 31 patients did not meet the inclusion criteria; not clear cell mRCC: n= 17, WHO performance state >2: n= 6, brain metastasis: n= 1, deep vein thrombosis: n= 1, no available biopsy: n= 2, impaired kidney function: n= 1, died during screening: n= 1, included in other study: n= 1.

Analysed (n= 46)
♦ Excluded from analysis (n= 0)

Lost to follow-up (n= 0)

Discontinued intervention (n= 0)

Allocation

Analysis

Follow-Up

Allocated to sunitinib (n= 46)

♦ Received allocated intervention (n= 46)

♦ Did not receive allocated intervention (n= 0)

Excluded (n= 31 )

♦  Not meeting inclusion criteria (n= 31)

♦  Declined to participate (n= 0)

Enrollment

Assessed for eligibility (n= 77 )
