# Supplementary material for: Predictive value of C-reactive protein in patients treated with sunitinib for metastatic clear cell renal cell carcinoma
Source: BMC Urol. 2017 Aug 31;17:74. doi: 10.1186/s12894-017-0267-6 (PMC5580299; doi:10.1186/s12894-017-0267-6)
Supplement: Supplementary file 2 — Inclusion criteria. (DOCX 17 kb) [file 12894_2017_267_MOESM2_ESM.docx]

**Table S1 – Inclusion criteria**

|  |
| --- |
| Metastatic or unresectable primary tumor of clear cell renal cell carcinoma |
| WHO performance status 0-2 |
| Non-earlier cytokine treatment or other cancer treatment |
| Radiation therapy against symptom given metastasis was possible, but not against lesion that we would like to evaluate response |
| Non symptomatic brain metastasis, but radiation treated brain metastasis where corticosteroids is stopped is allowed |
| >12 months since coronar bypass operation |
| >18 years |
| Not pregnant or breast feeding |
| Clinically or radiographic measurable disease according to the RECIST |
| >21 days since major surgery or damage |
| >2 days since biopsy, FNAC or central venous catheter |
| No ongoing grade 3 bleeding |
| None of the following the last 12 months: |
| -Myocardial infarction |
| -Serious unstable angina pectoris |
| -Symptomatic heart failure (not including EF <50% or >20% reduction of EF  compared to beginning of treatment) |
| -Stroke including TIA |
| -Pulmonary embolism |
| No other active malignant disease or not been treated for other cancers the last five years |
| Uncontrolled hypertension |
| Uncontrolled arrhythmia, especially prolonged QT-interval and bradycardias |
| Laboratory values of: |
| -Granulocytes >1,5 x 10^9/L |
| -Platelets >100 x 10^9/L |
| -Bilirubin and <1,5x upper normal limit |
| -ASAT <2,5x upper normal limit |
| -ALAT <2,5x upper normal limit |
| -Creatinine <1.5x upper normal limit  -International Normalized Ratio (INR) < 1,5x upper normal limit |
| No other compliance that could make the patient unsuitable to be included in a research protocol |
| Written consent from every patient |
|  |
| Abbrevations: WHO: World Health Organisation, RECIST: Response Evaluation Criteria in Solid Tumours, FNAC: Fine-Needle Aspiration Cytology, EF: Ejection Fraction, TIA: Transient Ischemic Attack, ASAT: aspartate aminotransferase, ALAT: alanine aminotransferase. |
