# Supplementary material for: Predictive value of C-reactive protein in patients treated with sunitinib for metastatic clear cell renal cell carcinoma
Source: BMC Urol. 2017 Aug 31;17:74. doi: 10.1186/s12894-017-0267-6 (PMC5580299; doi:10.1186/s12894-017-0267-6)
Supplement: Supplementary file 3 — Correlations between CRP and other variables. (DOCX 15 kb) [file 12894_2017_267_MOESM3_ESM.docx]

**Table S2.** Correlations between CRP and other variables

| **Variable** | **CRP at baseline** | |
| --- | --- | --- |
|  | Corr. coeff | *p*-value^1^ |
| Age | -0.38 | 0.01 |
| IMDC risk score | 0.61 | <0.001 |
| NLR baseline | 0.19 | 0.24 |
| Sum function score | -0.38 | 0.01 |
| Sum symptom score | 0.35 | 0.02 |
| S-Platelets | 0.45 | 0.002 |
| S-Creatinine | -0.53 | <0.001 |
| S-Hemoglobin | -0.59 | <0.001 |
| S-Calcium | 0.47 | 0.002 |
| S-Albumin | -0.74 | <0.001 |
| Tumor load | 0.35 | 0.02 |
|  | Median | *p*-value^2^ |
| Sex |  | 0.60 |
| Male | 17 mg/L |  |
| Female | 16 mg/L |  |
| WHO performance status |  | 0.008 |
| 0 | 9 mg/L |  |
| 1 | 78 mg/L |  |

^1^Spearman correlation. ^2^Mann Whitney test. Abbreviations: IMDC: International Metastatic Renal Cell Carcinoma Database Consortium. NLR: Neutrophil/ Lymphocyte Ratio.
