# Supplementary material for: Predictive value of C-reactive protein in patients treated with sunitinib for metastatic clear cell renal cell carcinoma
Source: BMC Urol. 2017 Aug 31;17:74. doi: 10.1186/s12894-017-0267-6 (PMC5580299; doi:10.1186/s12894-017-0267-6)
Supplement: Supplementary file 4 — Adverse effects. (DOCX 20 kb) [file 12894_2017_267_MOESM4_ESM.docx]

**Table S3. Adverse effects**

|  | Total | Grade 1+2 | Grade 3+4 |
| --- | --- | --- | --- |
| Laboratory |  |  |  |
| Hemoglobin | 22(50.0) | 22 (47.8) | 1 (2.2) |
| Lymphopenia | 4 (8.7) | 2 (4.3) | 2 (4.3) |
| Neutropenia | 11 (23.9) | 10 (21.7) | 1 (2.2) |
| Platelets | 14 (30.4) | 7 (15.2) | 7 (15.2) |
| Cardiac |  |  |  |
| Hypertension | 13 (28.3) | 4 (8.7) | 9 (19.6) |
| Hypertension (prior to 12 weeks) | 7 (15.2) | 2 (4.3) | 5 (10.9) |
| Left ventricular systolic  dysfunction | 2 (4.3) | 1 (2.2) | 1 (2.2) |
| Constitutional symptoms |  |  |  |
| Fatigue | 28 (60.9) | 21 (45.7) | 7 (15.2) |
| Fever | 3 (6.5) | 2 (4.3) | 1 (2.2) |
| Weight loss | 3 (6.5) | 3 (6.5) | 0 (0) |
| Dermatology/skin |  |  |  |
| Desquamation | 9 (19.6) | 9 (19.6) | 0 (0) |
| Hand-foot skin reaction | 17 (37.0) | 12 (26.1) | 5 (10.9) |
| Hypopigmentation | 7 (15.2) | 7 (15.2) | 0 (0) |
| Rash (other) | 5 (10.9) | 5 (10.9) | 0 (0) |
| Endocrine |  |  |  |
| Thyroid function (high) | 2 (4.3) | 2 (4.3) | 0 (0) |
| Thyroid function (low) | 9 (19.6) | 9 (19.6) | 0 (0) |
| Gastrointestinal |  |  |  |
| Anorexia | 8 (17.4) | 8 (17.4) | 0 (0) |
| Constipation | 4 (8.7) | 4 (8.7) | 0 (0) |
| Dehydration | 3 (6.5) | 2 (4.3) | 1 (2.2) |
| Diarrhea | 23 (0.5) | 18 (39.1) | 5 (10.9) |
| Dry mouth | 2 (4.3) | 2 (4.3) | 0 (0) |
| Gingiva | 2 (4.3) | 2 (4.3) | 0 (0) |
| Heartburn | 8 (17.4) | 6 (13.0) | 2 (4.3) |
| Mucositis (clinical exam) | 12 (26.1) | 11 (23.9) | 1 (2.2) |
| Mucositis (symptomatic/functional) | 10 (21.7) | 10 (21.7) | 0 (0) |
| Nausea | 24 (52.2) | 24 (52.2) | 0 (0) |
| Taste alteration (dysgeusia) | 12 (26.1) | 12 (26.1) | 0 (0) |
| Vomiting | 6 (13.0) | 5 (10.9) | 1 (2.2) |
| Infection |  |  |  |
| Infections | 7 (15.2)* | 5 (10.9) | 1 (2.2) |
| Lymphatics |  |  |  |
| Periorbital edema | 3 (6.5) | 3 (6.5) | 0 (0) |
| Neurology |  |  |  |
| Dizziness | 2 (4.3) | 2 (4.3) | 0 (0) |
| Mood alteration, depression | 2 (4.3) | 2 (4.3) | 0 (0) |
| Neuropathy, motor | 3 (6.5) | 0 (0) | 3 (6.5) |
| Ocular/visual |  |  |  |
| Watery eye | 3 (6.5) | 3 (6.5) | 0 (0) |
| Pain |  |  |  |
| Gastrointestinal pain | 12 (26.1) | 11 (23.9) | 1(2.2) |
| General pain | 3 (6.5) | 3 (6.5) | 0 (0) |
| Headache | 3 (6.5) | 3 (6.5) | 0 (0) |
| Musculoskeletal pain | 11 (23.9) | 8 (17.4) | 3 (6.5) |
| Pulmonary/upper respiration |  |  |  |
| Dyspnea | 2 (4.3) | 1 (2.2) | 1 (2.2) |
| Renal |  |  |  |
| Cystitis | 2 (4.3) | 2 (4.3) | 0 (0) |
| *One with Grade 5 (death due to appendicitis) | |  |  |
