# Supplementary material for: Predictive value of C-reactive protein in patients treated with sunitinib for metastatic clear cell renal cell carcinoma
Source: BMC Urol. 2017 Aug 31;17:74. doi: 10.1186/s12894-017-0267-6 (PMC5580299; doi:10.1186/s12894-017-0267-6)
Supplement: Supplementary file 5 — Summary of quality of life (QoL) scores. (DOCX 14 kb) [file 12894_2017_267_MOESM5_ESM.docx]

**Table S4.** The table shows the score (mean±SEM) of the global health status/ QoL, functional scales and symptom scales according to the EORTC QLQ-C30 scoring manual at baseline and after 12 weeks. The p-value is based on the non-parametric Wilcoxon signed rank sum test for the 28 patients evaluated at both time points.

|  | Baseline  (n=45) | 12 weeks  (n=28) | p-value |
| --- | --- | --- | --- |
|  |  |  |  |
| **Global health status / QoL** | 63±3 | 63±5 | 0.447 |
| Functional sum score | 75±3 | 77±4 | 0.219 |
| Symptom sum score | 20±3 | 22±4 | 0.166 |
|  |  |  |  |
| **Functional Scales** |  |  |  |
| Physical function | 73±3 | 74±5 | 0.977 |
| Role function | 62±5 | 70±6 | 0.138 |
| Emotional function | 77±3 | 81±4 | 0.415 |
| Cognitive function | 89±2 | 88±3 | 0.064 |
| Social function | 72±4 | 72±5 | 0.565 |
|  |  |  |  |
| **Symptom Scales** |  |  |  |
| Fatigue | 34±4 | 39±5 | 0.041* |
| Nausea / vomiting | 9±3 | 9±3 | 0.468 |
| Pain | 32±5 | 32±5 | 0.705 |
| Dyspnoea | 20±4 | 19±5 | 0.565 |
| Insomnia | 22±4 | 24±6 | 0.554 |
| Appetite loss | 16±4 | 24±5 | 0.110 |
| Constipation | 19±4 | 23±7 | 0.233 |
| Diarrhorea | 15±3 | 21±5 | 0.364 |
| Financial problems | 10±3 | 12±5 | 1.000 |
